# Transthyretin amyloid cardiomyopathy disease burden quantified using ^99m^Tc-pyrophosphate SPECT/CT: volumetric parameters versus SUVmax ratio at 1 and 3 hours

**DOI:** 10.1007/s12350-023-03353-w

**Published:** 2023-08-21

**Authors:** Satoru Watanabe, Kenichi Nakajima, Fumihito Toshima, Hiroshi Wakabayashi, Shohei Yoshida, Hiroto Yoneyama, Junji Komatsu, Takahiro Konishi, Seigo Kinuya

**Affiliations:** 1 Department of Functional Imaging and Artificial Intelligence, Kanazawa University, Kanazawa, Japan; 2 Department of Nuclear Medicine, Kanazawa University Hospital, 13-1 Takara-machi, Kanazawa, 920-8641 Japan; 3 Department of Radiology, Kanazawa University Graduate School of Medical Sciences, Kanazawa, Japan; 4 Department of Cardiovascular Medicine, Kanazawa University Graduate School of Medical Sciences, Kanazawa, Japan; 5 Department of Radiological Technology, Kanazawa University Hospital, Kanazawa, Japan; 6 Department of Neurology and Neurobiology of Aging, Kanazawa University Graduate School of Medical Sciences, Kanazawa, Japan

**Keywords:** Amyloid heart disease, cardiomyopathy, heart failure, SPECT, hybrid imaging, multimodality

## Abstract

**Background:**

Various parameters derived from technetium-99m pyrophosphate (^99m^Tc-PYP) single-photon emission computed tomography (SPECT) correlate with the severity of transthyretin amyloid cardiomyopathy (ATTR-CM). However, the optimal metrics and image acquisition timing required to quantify the disease burden remain uncertain.

**Methods and results:**

We retrospectively evaluated ^99m^Tc-PYP SPECT/CT images of 23 patients diagnosed with ATTR-CM using endomyocardial biopsies and/or gene tests. All patients were assessed by SPECT/CT 1 hour after ^99m^Tc-PYP injection, and 13 of them were also assessed at 3 hours. We quantified ^99m^Tc-PYP uptake using the volumetric parameters, cardiac PYP volume (CPV) and cardiac PYP activity (CPA). We also calculated the SUVmax ratios of myocardial SUVmax/blood pool SUVmax, myocardial SUVmax/bone SUVmax, and the SUVmax retention index. We assessed the correlations between uptake parameters and the four functional parameters associated with prognosis, namely left ventricular ejection fraction, global longitudinal strain, myocardial extracellular volume, and troponin T. CPV and CPA correlated more closely than the SUVmax ratios with the four prognostic factors. Significant correlations between volumetric parameters and prognostic factors were equivalent between 1 and 3 hours.

**Conclusions:**

The disease burden of ATTR-CM was quantified more accurately by volumetric evaluation of ^99m^Tc-PYP SPECT/CT than SUVmax ratios and the performance was equivalent between 1 and 3 hours.

**Supplementary Information:**

The online version contains supplementary material available at 10.1007/s12350-023-03353-w.

## Introduction

Transthyretin amyloid cardiomyopathy (ATTR-CM) is an increasingly recognized cause of heart failure (HF) resulting from the myocardial deposition of misfolded protein fibrils.^1^ Disease-modifying therapies for ATTR-CM, ATTR stabilizer and gene silencing pharmacotherapy have recently been developed.^2,3^ This has resulted in a clinical need for early detection and accurate quantitation of disease burden, prognosis, and response to treatment. ATTR-CM can be non-invasively diagnosed in patients without light-chain cardiac amyloidosis using bone scintigraphy with technetium-99m pyrophosphate (^99m^Tc-PYP), ^99m^Tc-3,3-diphosphono-1,2-propanodicarboxylic acid (DPD), or ^99m^Tc-hydroxymethylenediphosphonate.^4,5^ Recently, bone scintigraphy has been actively investigated not only for diagnosis, but also for assessments of cardiac function, disease burden, prognosis, and response to therapy.^6–19^

Various quantitative parameters derived from ^99m^Tc-PYP scintigraphic images using three-dimensional single-photon emission computed tomography (SPECT) are useful for assessing the ATTR-CM disease burden and prognosis.^6–14^ Accurate quantitation also enables interval monitoring of disease progression and responses to treatment. However, an optimal method has not been established. Uptake parameters such as the standardized uptake value (SUV), the SUV ratio (normalized SUV and the SUV retention index), as well as volumetric parameters have been investigated using images acquired at various timing. Most studies have investigated ^99m^Tc-PYP SPECT images acquired 3 hours after radiotracer injection to minimize the influence of relatively high blood pool activity at 1 hour.^6–11^ Blood pool activity is particularly important for patients without ATTR-CM because it can result in false-positive diagnoses. However, the optimal method for diagnosis, including cohorts without ATTR-CM, is not necessarily the same as that for assessing disease burden.^14^ This should be determined based on cohorts with ATTR-CM.^6,11,17–20^ Moreover, images acquired at 1 hour after radiotracer injection have a unique feature that is lacking in images acquired at 3 hours; myocardial abnormal uptake on bone scintigraphic images peaks at 1 hour, then slowly declines.^20^

The aim of this study was to define whether ^99m^Tc-PYP volumetric parameters or SUVmax ratios are more effective for quantifying ATTR-CM disease burden at 1 and 3 hours after radiotracer injection.

## Materials and methods

### Study population

We retrospectively evaluated patients who were assessed by ^99m^Tc-PYP SPECT/CT at Kanazawa University Hospital between September 2019 and February 2023 and diagnosed with ATTR-CM from endomyocardial biopsies (EMBs) and/or TTR gene tests. The diagnostic criteria for ATTR-CM were based on one or more of the following: (1) EMB positive for ATTR and (2) having a documented TTR genetic mutation and evidence of cardiomyopathy without apparent plasma cell dyscrasia (serum and urine immunofixation and serum-free light-chain assays). When patients had undergone multiple ^99m^Tc-PYP tests during follow-up, only one was included in the present study. The ethics committee at Kanazawa University approved this study and waived the requirement for written informed consent because the study retrospectively selected patient data.

### Imaging acquisition

Patients were injected intravenously with ~ 740 MBq (20 mCi) of ^99m^Tc-PYP. Thorax planar images were acquired for 2 min at 1 and 3 hours later using a hybrid SPECT/CT system (Symbia Intevo, Siemens Medical Solutions AG, Erlangen, Germany) with a low-energy high-resolution collimator. The acquisition time of 2 minutes was acceptable for 750,000 counts in Japanese patients. Before November 2021, SPECT/CT images of the thorax were acquired only 1 hour after radiotracer injection. Thereafter, SPECT/CT images were acquired at both 1 and 3 hours after injection. The SPECT/CT parameters comprised step-and-shoot acquisition with body-contour non-circular orbit, 120 steps at 15 s/each with a total acquisition time of 20 minutes, and zoom 1.0.

Images were reconstructed to a 128 × 128 matrix using a dedicated iterative algorithm (xSPECT QUANT) with 72 iterations and 1 subset, and a 10-mm Gaussian filter. A low-dose, free-breathing, and non-contrast CT image was acquired for attenuation correction and anatomical localization using the following parameters: 130 kV, 50 mAs with care dose, pitch 1.5, rotation time .6 s, collimation 16 × 1.2. The CT-based attenuation and scatter corrections were performed automatically.

### Quantitative interpretation of images

**Figure 1:**
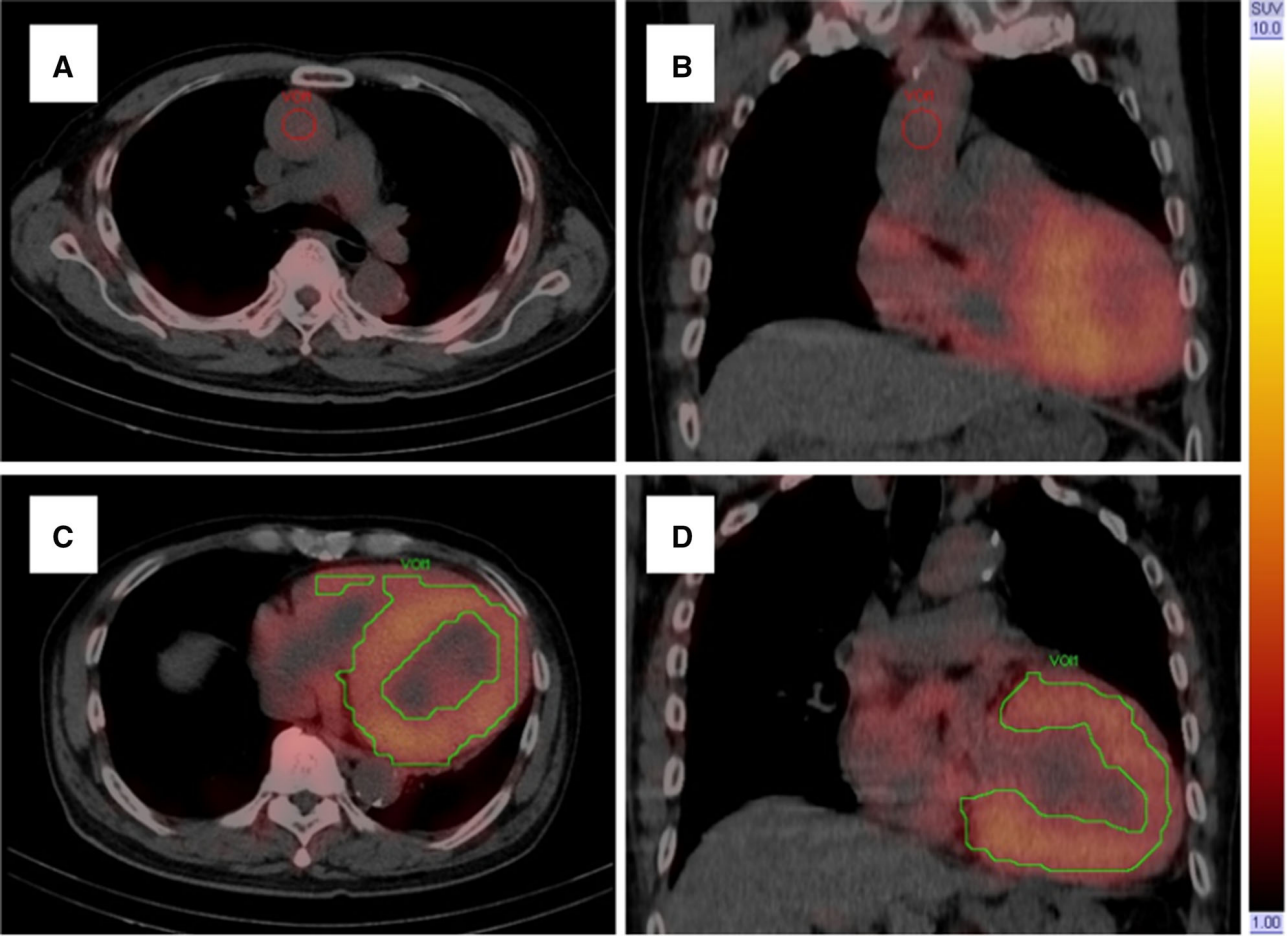
An example of SUV-threshold volumetric evaluation of ^99m^Tc-PYP SPECT/CT at 1 h after radiotracer injection. (A, B) Red circles is spherical volume of interest placed in center of ascending aortic blood pool. (C, D) Green contours indicate myocardial regions with ^99m^Tc-PYP uptake > 1.4 × aortic blood pool radioactivity (SUVmax) automatically evaluated using xSPECT Quant. ^*99m*^*Tc-PYP*, Technetium-99m pyrophosphate; *SPECT*, single-photon emission computed tomography; *SUV*, standardized uptake value

Figure 1 shows an example of SPECT/CT volumetric evaluation. We used xSPECT Quant (Siemens) to calculate SUVmax, SUVmean, and volumetric parameters. We measured ^99m^Tc-PYP activity in an aortic blood pool using SUVmax. A spherical volume of interest (VOI; diameter equivalent to half that of the aorta) was positioned in the center of the ascending aorta at the level of the pulmonary artery bifurcation on fused SPECT/CT images.^10,21^ A polygonal VOI was manually placed to include uptake by the left and right ventricles, but not adjacent ribs and the sternum.^8–10^ Total volumes of voxels in the myocardial regions with ^99m^Tc-PYP uptake > 1.2, 1.4, and 1.6 × the SUVmax of the aortic blood pool were automatically evaluated using xSPECT Quant and defined as cardiac PYP volumes (CPV1.2, 1.4, and 1.6).^10^ We visually confirmed that the abnormal uptake areas were in the myocardial regions. The threshold values of 1.2, 1.4, and 1.6 were based on our previous study.^10^ We also defined cardiac PYP activity (CPA) as CPV × (myocardial SUVmean/aortic blood pool SUVmax), using SUVmean in myocardial regions with uptake > 1.2, 1.4, and 1.6 × aortic blood pool SUVmax (CPA1.2, 1.4, and 1.6).^8,9^ CPA reflects the volume and intensity of abnormal uptake. We used SUVmean, which reflects the average uptake in the abnormal uptake areas, to evaluate CPA.^8,9,14,22^

We also evaluated SUVmax within the entire left and right ventricular myocardium. We determined bone SUVmax by placing a spherical VOI at the center of an intact thoracic vertebral body (T12 unless identified as abnormal from a review of bone scan images).^21^ If T12 was not intact, the spherical VOI was placed at the nearest normal vertebral body. We then determined the soft-tissue SUVmax by positioning a spherical VOI at the paraspinal muscle near T12. Myocardial SUVmax/aortic blood pool SUVmax,^19,20,23^ myocardial SUVmax/vertebral SUVmax,^13,19,20,23,24^ and SUVmax retention index^16^ = (myocardial SUVmax/vertebral SUVmax) × paraspinal muscle SUVmax were calculated and we referred to them as SUVmax ratios.

We also evaluated the heart to contralateral lung (H/CL) ratio and the visual grading score.^5^ The H/CL ratio was calculated as the fraction of the average counts in a circular region of interest (ROI) drawn over the heart of a planar image to that in the contralateral lung ROI of the identical size. The visual grading score was determined by comparing myocardial uptake to rib uptake (grade 0, no uptake; grade 1, uptake less than rib; grade 2, uptake equal to rib; and grade 3, uptake greater than rib).

### Cardiovascular magnetic resonance (CMR)

We acquired CMR images using a 3.0-T MRI scanner (Ingenia; Siemens Medical Solutions AG). Native and postcontrast T1 maps were acquired using a modified look-locker technique (with a 5-3-3 beat scheme), under the following parameters: repetition time, shortest; echo time, shortest; flip angle, 20°; slice thickness, 10 mm; matrix, 160 × 160; field of view, 30 cm. Postcontrast T1 maps were scanned 7−10 minutes after 10 mL (1.0 mol/L) of Gadovist (Bayer Pharma AG, Berlin, Germany) was injected. The myocardial extracellular volume (ECV) was calculated as the ratio of changes in myocardial to blood relaxation rates and adjusted to the fractional blood volume of distribution (1—hematocrit). The T1 values were quantified on ROI from the myocardium (septal midventricular wall from the short-axis slice) and the blood (left ventricular blood pool) on both native and contrast T1 maps. The largest possible ROIs were manually drawn, avoiding regions of misregistration (cross-hatched areas) between images at each inversion time. We then applied a copy-and-paste technique to place the same shape ROIs at the exact location on native and postcontrast T1 maps. Thereafter, we calculated the ECV using the average value of each ROI.

### Other assessments

Left ventricular ejection fraction (LVEF) and global longitudinal strain (GLS) were assessed by echocardiography. High-sensitivity cardiac troponin T values, hematocrit (Ht), and estimated glomerular filtration rates (eGFR) were measured by blood examination.

### Analysis

We assessed the performance of quantifying disease burden. We evaluated correlations between uptake parameters (CPV, CPA, myocardial SUVmax, SUVmax ratios, H/CL ratio, and visual grading score at 1 and 3 hours) and functional parameters associated with prognosis (LVEF,^25^ GLS,^26,27^ ECV,^28^ and troponin T^11,25^).

### Statistical analysis

Continuous variables summarized as means ± standard deviation (SD) were compared using Student’s t-tests. Uptake parameters were compared between 1 and 3 hours using Student’s t-tests for paired comparisons. Categorical variables were summarized as numbers (%). Visual grading scores were compared between 1 and 3 hours using Wilcoxon signed-rank test. Correlations between parameters were assessed using Pearson correlation coefficients (Spearman rank-correlation coefficients for visual grading score). Interobserver variability was assessed using an intraclass correlation coefficient (ICC) and Bland-Altman analysis. Values with *P* < .05 were considered statistically significant. All data were analyzed using JMP^®^ Pro 17 (SAS Institute, Cary, NC, USA).

## Results

### Patient population

Table 1 summarizes the characteristics of all 23 patients (mean age, 78.0 ± 10.1 years; male, 18 [78%]). All patients were assessed by SPECT/CT 1 hour after ^99m^Tc-PYP injection. Thirteen of them were also assessed by SPECT/CT at 3 hours because we changed the imaging protocol in November 2021, and they included patients who had been diagnosed with hereditary ATTR-CM and treated with tafamidis (Table 1). All 23 patients were proven positive for ATTR amyloid from biopsy specimens. None of the image data have been previously published.

**Table 1:** Characteristics of the patients

	All (*n* = 23)	SPECT acquisition at 1 and 3 h (*n* = 13)	SPECT acquisition at only 1 h (*n* = 10)
Age (years)	78.0 ± 10.1	75.9 ± 12.2	80.9 ± 6.0
Male	18 (78%)	10 (77%)	8(80%)
ATTR-CM type			
ATTRwt-CM	18 (78%)	8 (62%)	10(100%)
ATTRv-CM	5 (22%)	5 (38%)	0(0%)
Tafamidis			
Before	18 (78%)	8 (62%)	10(100%)
During	5 (22%)^a^	5 (38%)^a^	0(0%)
TTR assessment			
Endomyocardial biopsy	22 (96%)^b^	12 (92%)^b^	10 (100%)
TTR gene tests	23 (100%)	13 (100%)	10 (100%)
LVEF (%)	53 ± 14 (*n* = 22)	58 ± 16 (*n* = 12)	47 ± 9 (*n* = 10)
GLS (%)	− 10.3 ± 2.9 (*n* = 13)	− 10.5 ± 3.7 (*n* = 6)	− 10.1 ± 2.2 (*n* = 7)
ECV (%)	54.4 ± 8.8 (*n* = 12)	50.5 ± 5.8 (*n* = 8)	62.2 ± 9.0 (*n* = 4)
Troponin T (ng/mL)	.07 ± .04 (*n* = 18)	.06 ± .03 (*n* = 9)	.08 ± .04 (*n* = 9)
eGFR (mL/min/1.73m^2^)	55 ± 19 (*n* = 23)	59 ± 19 (*n* = 13)	49 ± 20 (*n* = 10)

Continuous data are shown as means ± standard deviation. Categorical data are shown as n (%)

*ATTR-CM*, transthyretin amyloid cardiomyopathy; *ATTRv*, hereditary ATTR; *ATTRwt*, wild type ATTR; *ECV*, extracellular volume; *eGFR*, estimated glomerular filtration rate; *GLS*, global longitudinal strain; *LVEF*, left ventricular ejection fraction; *SPECT*, single-photon emission computed tomography; *TTR*, transthyretin

^a^All patients with ATTRv-CM had already been treated with tafamidis

^b^One patient did not undergo a myocardial biopsy, but an intestinal biopsy was positive for ATTR amyloid

### Quantitative uptake parameters

**Figure 2:**
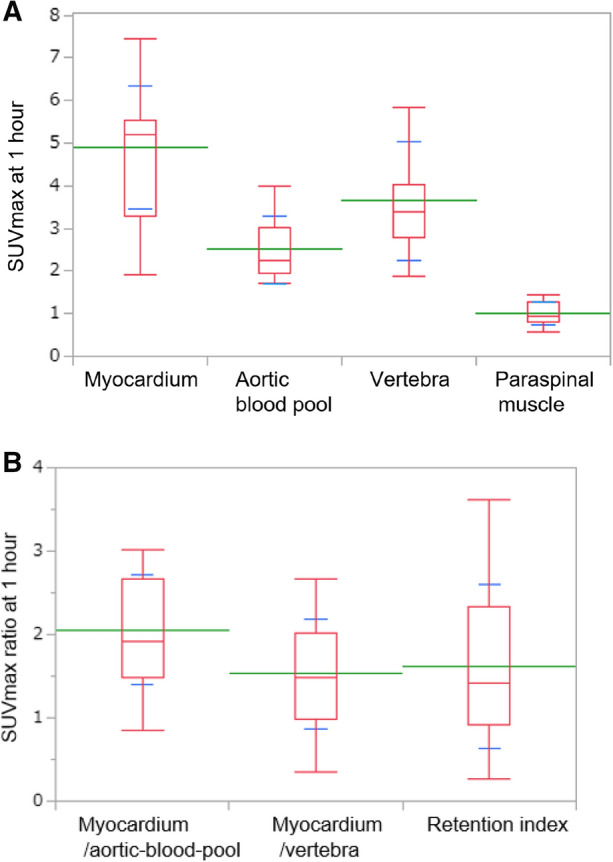
Comparison of SUVmax (A) and SUVmax ratios (B) at 1 h after ^99m^Tc-PYP injection (*n* = 23). Green and blue lines indicate means and standard deviations, respectively. Box plot indicates medians, 25%, and 75% quartiles with whiskers at both ends. Retention index = (myocardial SUVmax/vertebral SUVmax) × paraspinal muscle SUVmax. ^*99m*^*Tc-PYP*, Technetium-99m pyrophosphate; *SUV*, standardized uptake value

Table 2 and Figure 2 summarize the uptake parameters in images of all 23 patients acquired 1 hour after ^99m^Tc-PYP injection. The volumetric parameters CPV and CPA decreased as thresholds increased. The myocardial SUVmax/aortic blood pool SUVmax and myocardial SUVmax/vertebral SUVmax were 2.1 ± .7 and 1.5 ± .7, respectively. In a patient with low abnormal myocardial uptake, we evaluated SUVmax in the interventricular septum on fused SPECT/CT images.

**Table 2:** Uptake parameters at 1 h after ^99m^Tc-PYP injection

	All patients (*n* = 23)
Volumetric parameters (cm^3^)	
CPV1.2	203 ± 193
CPV1.4	144 ± 157
CPV1.6	100 ± 118
CPA1.2	343 ± 345
CPA1.4	265 ± 300
CPA1.6	198 ± 240
SUVmax	
Myocardium	4.9 ± 1.4
Aortic blood pool	2.5 ± .8
Vertebra	3.6 ± 1.3
Paraspinal muscle	1.0 ± .3
SUVmax ratio	
Myocardium/aortic blood pool	2.1 ± .7
Myocardium/vertebra	1.5 ± .7
Retention index^a^	1.6 ± 1.0
H/CL ratio	1.8 ± .3
Visual grading score	
Grade 1	2 (9%)^b^
Grade 3	21 (91%)

*CPV*, cardiac pyrophosphate volume; *CPA*, cardiac pyrophosphate activity; *SUV*, standardized uptake value; *H/CL*, heart to contralateral lung

^a^Retention index = (myocardial SUV/vertebral SUV) × paraspinal muscle SUV

^b^One patient had Val30Met mutation

Interobserver variability was assessed by SW and TK for CPV1.4 at 1 hour in all 13 patients with SPECT/CT images acquired at 1 and 3 hours. The ICC(2,1) was excellent at .990, and the interobserver variability was low (mean difference 8.3 cm^3^, 95% limits of agreement -4.2 to 20.9 cm^3^, *P* = n.s., Supplemental Figure 1).

### Other assessments

Table 1 summarizes the values for LVEF, GLS, ECV, troponin T, and eGFR. We assessed the LVEF in 22 patients, GLS in 13, and ECV in 12 at 5.8 ± 52.3, 5.8 ± 68.3, and 5.8 ± 69.7 days, respectively, before ^99m^Tc-PYP SPECT/CT. Ht was measured within 24 hours of CMR. We assessed troponin T in 18 patients and eGFR in 23 at 8.6 ± 32.8 days after, and 2.7 ± 5.7 days before ^99m^Tc-PYP SPECT/CT, respectively. LVEF and ECV differed significantly between the two patient groups, but GLS, troponin T, and eGFR did not.

### Correlations with prognostic factors

**Figure 3:**
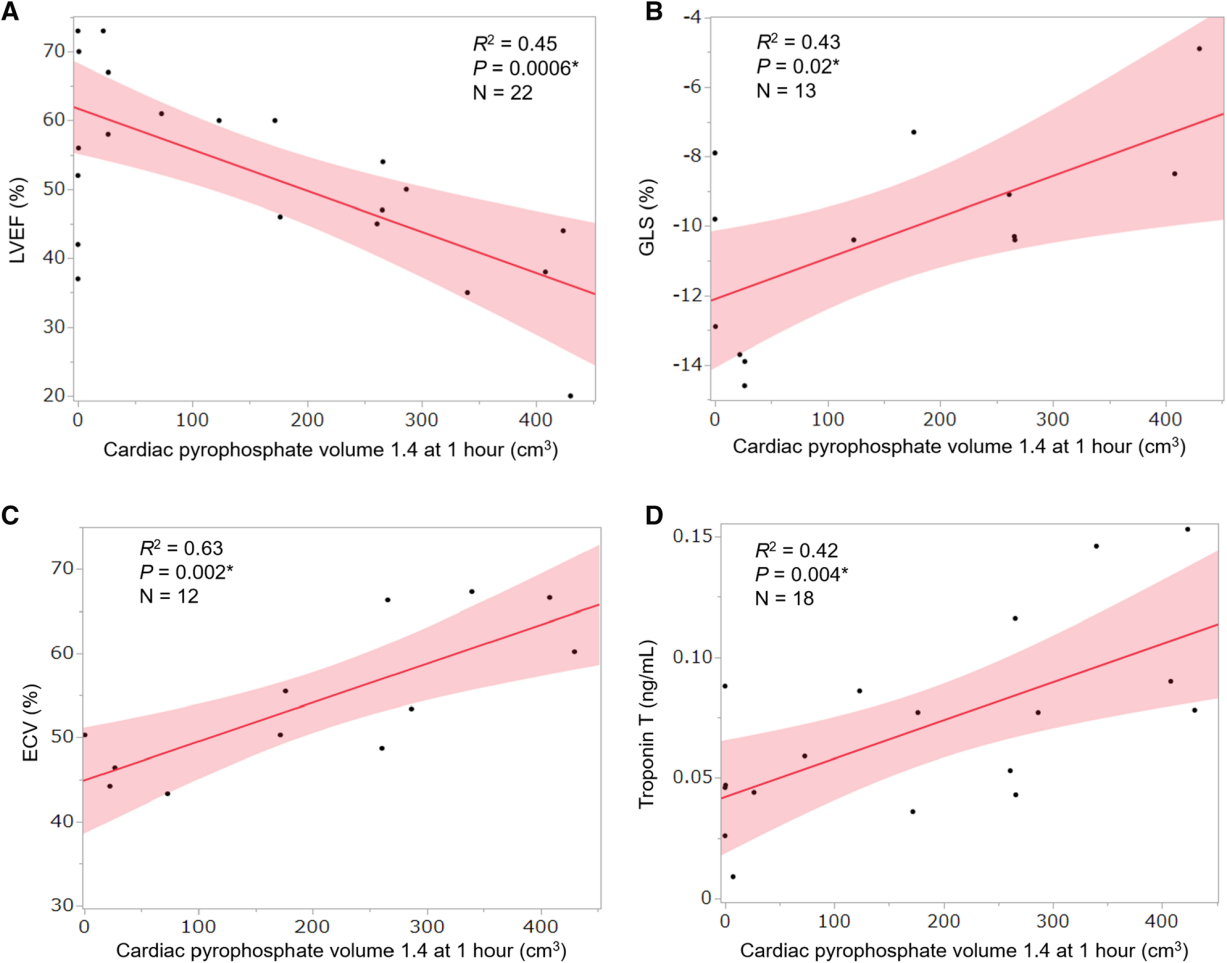
Representative correlations between CPV at 1 h and four prognostic factors in patients with ATTR-CM (*n* = 23). Solid lines, values predicted from linear regression analysis; shaded bands, 95% confidence intervals. *ATTR-CM*, transthyretin amyloid cardiomyopathy; *CPV*, cardiac pyrophosphate volume; *ECV*, extracellular volume; *GLS*, global longitudinal strain; *LVEF*, left ventricular ejection fraction; *R*, Pearson correlation coefficient. *P < .05

Table 3 summarizes correlations between uptake parameters at 1 hour and the four functional parameters associated with prognosis in all 23 patients. Figure 3 shows representative correlations between CPV and the four prognostic factors. Table 3 shows closer correlations in the same order of volumetric parameters (CPV1.4 and CPA1.4) > SUVmax ratios (myocardial SUVmax/aortic blood pool SUVmax, myocardial SUVmax/vertebral SUVmax, and SUVmax retention index) > myocardial SUVmax for the four prognostic factors, LVEF, GLS, ECV, and troponin T.

**Table 3:** Correlations between uptake parameters at 1 h and four prognostic factors

	LVEF (*n* = 22)		GLS (*n* = 13)		ECV (*n* = 12)		Troponin T (*n* = 18)	
	*R*^2^	*P*	*R*^2^	*P*	*R*^2^	*P*	*R*^2^	*P*
Volumetric parameters								
CPV1.4	.45	.0006*	.43	.02*	.63	.002*	.42	.004*
CPA1.4	.45	.0006*	.43	.01*	.63	.002*	.42	.004*
SUVmax ratio								
Myocardium/aorta	.26	.0155*	.17	.16	.58	.004*	.26	.032*
Myocardium/vertebra	.29	.0093*	.11	.27	.09	.352	.04	.405
Retention index^a^	.40	.0016*	.25	.08	.06	.439	.05	.359
Myocardial SUVmax	.14	.0834	.01	.73	.32	.056	.00	.876
H/CL ratio	.13	.1005	.07	.38	.64	.002*	.12	.167
Visual grading score	.01	.7405	.10	.30	–^b^	–^b^	.04	.402

*CPA*, cardiac pyrophosphate activity; *CPV*, cardiac pyrophosphate volume; *ECV*, extracellular volume fraction; *GLS*, global longitudinal strain; *H/CL*, heart to contralateral lung; *LVEF*, left ventricular ejection fraction; *R*, correlation coefficient; *SUV*, standardized uptake value

*P < .05

^a^Retention index = (myocardial SUV/vertebral SUV) × paraspinal muscle SUV

^b^Visual grading score of all 12 patients were 3

Both CPV1.4 and CPA1.4 significantly correlated with the four prognostic factors (*r*^2^ = .42−.63, *P* = .0006−.02), whereas SUVmax ratios did not significantly correlate with GLS. Myocardial SUVmax/vertebral SUVmax and the SUVmax retention index did not significantly correlate with ECV and troponin T. Myocardial SUVmax did not significantly correlate with any of the prognostic factors (*r*^2^ = .00−.32, *P* = .056−.876). The H/CL ratio and visual grading score did not correlate with LVEF, GLS, and troponin T.

We also evaluated correlations between uptake parameters at 1 h and four prognostic factors in 18 patients not treated with tafamidis. Closer correlations were observed in the same order of volumetric parameters, SUVmax ratios, myocardial SUVmax for the four prognostic factors in 18 patients not treated with tafamidis (Supplemental Table 1). The H/CL ratio and visual grading score did not correlate significantly with LVEF, GLS, and troponin T.

We evaluated a CPV1.4 using only the left ventricular region (CPV1.4_LV_), excluding right ventricular activity. The CPV1.4 and CPV1.4_LV_ similarly correlated with the four prognostic factors (Supplemental Table 2). Seven patients (30.4%) had right ventricular uptake of > 1.4 × the SUVmax of the aortic blood pool 1 hour after radiotracer injection.

The uptake parameters did not significantly correlate with eGFR (Supplemental Table 3).

### Time dependence of uptake parameters and correlations

**Figure 4:**
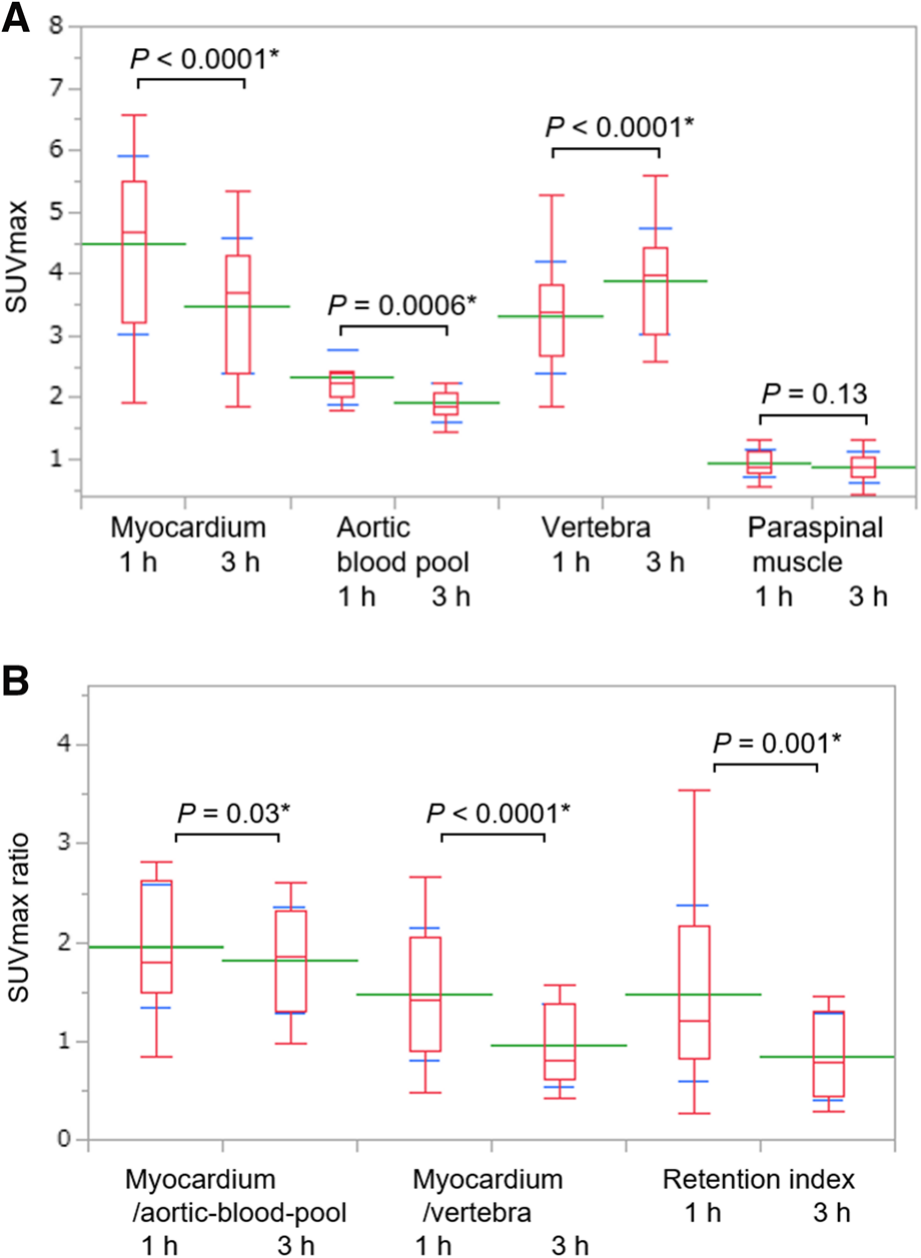
Comparison of parameters between 1 and 3 h after injecting ^99m^Tc-PYP into 13 patients with ATTR-CM who were assessed twice. (A) Myocardial SUVmax, aortic blood pool SUVmax, vertebral SUVmax, and paraspinal muscle SUVmax, and (B) myocardial SUVmax/aortic blood pool SUVmax, myocardial SUVmax/vertebral SUVmax, and SUVmax retention index. Green and blue lines indicate means and standard deviations, respectively. Box plot indicates medians, 25%, and 75% quartiles with whiskers at both ends. *SUV*, standardized uptake value. *P < .05

Table 4 and Figure 4 summarize the uptake parameters of the 13 patients who were assessed by ^99m^Tc-PYP SPECT/CT at 1 and 3 hours after ^99m^Tc-PYP injection. The CPV1.4 and 1.6, CPA, myocardial SUVmax, aortic blood pool SUVmax, SUVmax ratios, H/CL ratio, and visual grading score were significantly higher at 1 hour than at 3 hours. In contrast, vertebral SUVmax was significantly higher at 3 hours than at 1 hour. Paraspinal muscle SUVmax did not significantly differ between 1 and 3 hours.

**Table 4:** Uptake parameters of images acquired from patients (*n* = 13) at 1 and 3 h after ^99m^Tc-PYP injection

	1 h	3 h	*P* value
Volumetric parameters (cm^3^)			
CPV1.2	167 ± 173	141 ± 163	.05
CPV1.4	112 ± 142	84 ± 122	.02*
CPV1.6	76 ± 105	55 ± 91	.03*
CPA1.2	278 ± 307	220 ± 272	.02*
CPA1.4	206 ± 266	147 ± 219	.01*
CPA1.6	150 ± 211	102 ± 171	.02*
SUVmax			
Myocardium	4.5 ± 1.4	3.5 ± 1.1	< .0001*
Aortic blood pool	2.3 ± .4	1.9 ± .3	.0006*
Vertebra	3.3 ± .9	3.9 ± .9	< .0001*
Paraspinal muscle	.9 ± .2	.9 ± .3	.13
SUVmax ratio			
Myocardium/aortic blood pool	2.0 ± .6	1.8 ± .5	.03*
Myocardium/vertebra	1.5 ± .7	1.0 ± .4	< .0001*
Retention index^a^	1.5 ± .9	.8 ± .4	.001*
H/CL ratio	1.7 ± .3	1.6 ± .2	.001*
Visual grading score			
Grade 1	1 (8%)^b^	1 (8%)^b^	.02*
Grade 2	0 (0%)	6 (46%)	
Grade 3	12 (92%)	6 (46%)	

*CPA*, cardiac pyrophosphate activity; *CPV*, cardiac pyrophosphate volume; *H/CL*, heart to contralateral lung; *SUV*, standardized uptake value

*P < .05

^a^Retention index = (myocardial SUV/vertebral SUV) × paraspinal muscle SUV

^b^One patient had Val30Met mutation

**Figure 5:**
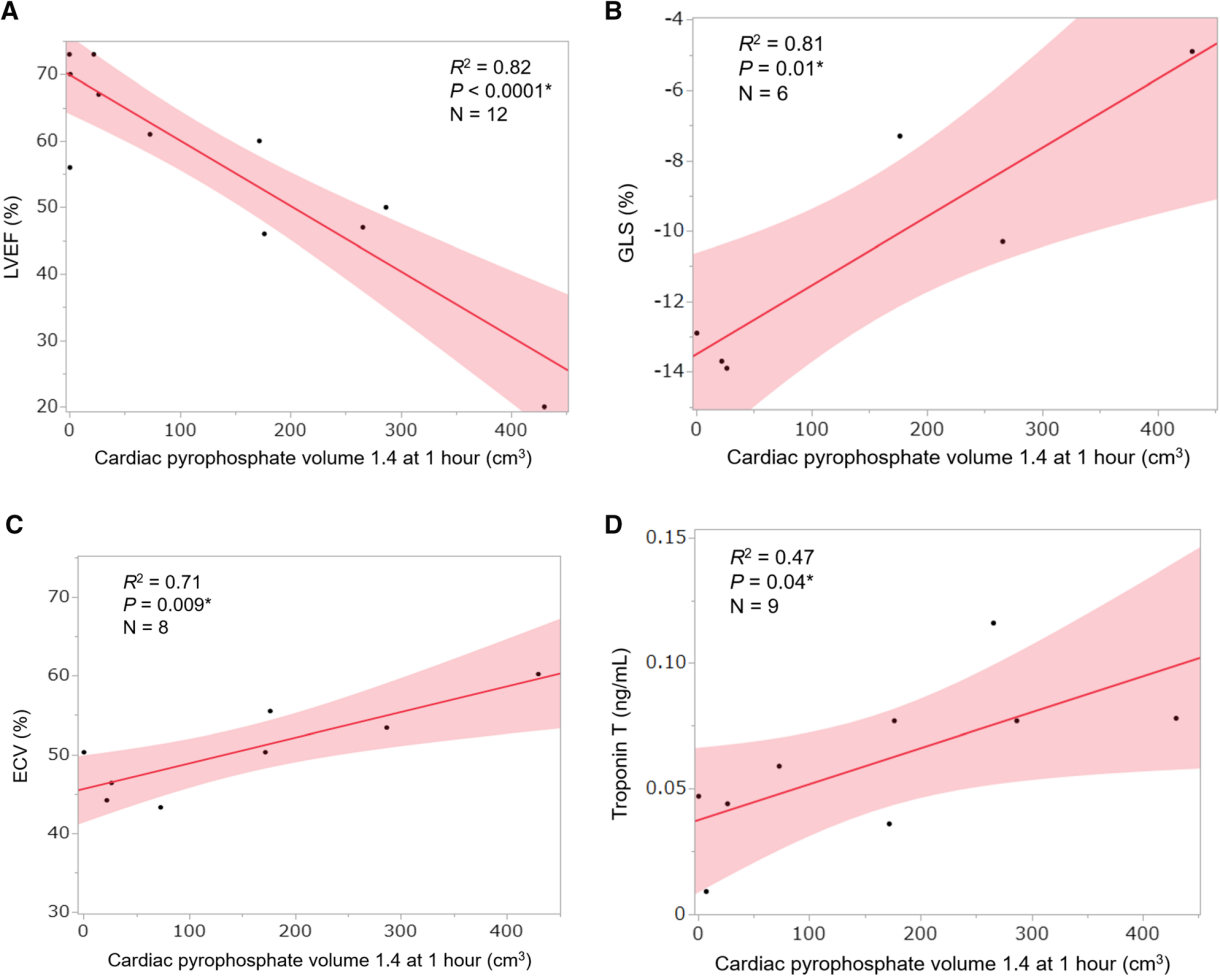
Representative correlations between CPV at 1 h and four prognostic factors in patients with SPECT/CT images acquired at 1 and 3 h (*n* = 13). Solid lines, values predicted from linear regression analysis; shaded bands, 95% confidence intervals. *ATTR-CM*, transthyretin amyloid cardiomyopathy; *CPV*, cardiac pyrophosphate volume; *ECV*, extracellular volume; *GLS*, global longitudinal strain; *LVEF*, left ventricular ejection fraction; *R*, Pearson correlation coefficient. *P < .05

Table 5 summarizes correlations between uptake parameters and the four prognostic factors in patients with SPECT/CT images acquired at 1 and 3 hours after ^99m^Tc-PYP injection. Figure 5 shows representative correlations between CPV and the four prognostic factors. Table 5 shows significant correlations between the volumetric parameters CPV1.4 and CPA1.4 and the four prognostic factors at 1 and 3 hours. Correlations between volumetric parameters and LVEF, ECV, or troponin T were equivalent between 1 and 3 hours. Correlations between volumetric parameters and GLS were slightly closer at 1 hour than at 3 hours in six patients. The SUVmax retention index closely correlated with LVEF, GLS, and ECV at 1 hour, but the correlation coefficients decreased at 3 hours.

**Table 5:** Correlations between uptake parameters and four prognostic factors in patients with SPECT/CT images acquired at 1 and 3 h

	LVEF (*n* = 12)		GLS (*n* = 6)		ECV (*n* = 8)		Troponin T (*n* = 9)	
	*R*^2^	*P*	*R*^2^	*P*	*R*^2^	*P*	*R*^2^	*P*
1 h								
Volumetric parameters								
CPV1.4	.82	< .0001*	.81	.01*	.71	.009*	.47	.042*
CPA1.4	.82	< .0001*	.83	.01*	.73	.007*	.46	.045*
SUVmax ratio								
Myocardium/aorta	.57	.0044*	.70	.04*	.56	.033*	.45	.047*
Myocardium/vertebra	.73	.0004*	.46	.14	.77	.004*	.28	.141
Retention index	.85	< .0001*	.86	.01*	.85	.001*	.38	.079
Myocardial SUVmax	.43	.0205*	.24	.33	.50	.049*	.14	.320
H/CL ratio	.17	.1841	.37	.20	.43	.075	.00	.962
Visual grading score	.15	.2028	–^a^	–^a^	–^a^	–^a^	–^a^	–^a^
3 h								
Volumetric parameters								
CPV1.4	.78	.0001*	.71	.04*	.70	.010*	.49	.03*
CPA1.4	.78	.0001*	.70	.04*	.69	.011*	.49	.04*
SUVmax ratio								
Myocardium/aorta	.64	.0019*	.55	.09	.57	.031*	.43	.06
Myocardium/vertebra	.58	.0039*	.45	.14	.63	.019*	.27	.14
Retention index	.48	.0126*	.42	.16	.46	.063	.68	.01*
Myocardial SUVmax	.43	.0213*	.29	.27	.47	.061	.26	.16
H/CL ratio	.22	.1211	.22	.35	.38	.101	.02	.74
Visual grading score	.77	.0002*	.69	.04*	.68	.012*	.21	.21

*CPA*, cardiac pyrophosphate activity; *CPV*, cardiac pyrophosphate volume; *ECV*, extracellular volume; *GLS*, global longitudinal strain; *H/CL*, heart to contralateral lung; *LVEF*, left ventricular ejection fraction; *R*, correlation coefficient; *SUV*, standardized uptake value

*P < .05

^a^ Visual grading score of all patients were 3 at 1h

### Threshold dependence of correlations

Table 6 summarizes the threshold dependence of correlations between volumetric parameters at 1 hour and the four prognostic factors in all 23 patients. The threshold dependence of correlations was low among six volumetric parameters, and all six significantly correlated with the four prognostic factors. CPV1.4 and CPA1.4 showed slightly higher correlation coefficients (*r*^2^ = .42−.63) than CPV1.2 and CPA1.2 (*r*^2^ = .37−.62).

**Table 6:** Correlations between volumetric parameters at 1 h and four prognostic factors

Volumetrics	LVEF (*n* = 22)		GLS (*n* = 13)		ECV (*n* = 12)		Troponin T (*n* = 18)	
	*R*^2^	*P*	*R*^2^	*P*	*R*^2^	*P*	*R*^2^	*P*
CPV1.2	.41	.0013*	.37	.03*	.61	.003*	.41	.005*
CPV1.4	.45	.0006*	.43	.02*	.63	.002*	.42	.004*
CPV1.6	.43	.0009*	.44	.01*	.65	.002*	.42	.004*
CPA1.2	.43	.0010*	.40	.02*	.62	.002*	.41	.004*
CPA1.4	.45	.0006*	.43	.01*	.63	.002*	.42	.004*
CPA1.6	.43	.0010*	.44	.01*	.64	.002*	.41	.004*

*CPA*, cardiac pyrophosphate activity; *CPV*, cardiac pyrophosphate volume; *ECV*, extracellular volume; *GLS*, global longitudinal strain; *LVEF*, left ventricular ejection fraction; *R*, Pearson correlation coefficient

*P < .05

Table 7 summarizes the threshold dependence of correlations between volumetric parameters and the four prognostic factors in patients with SPECT/CT images acquired at 1 and 3 hours after ^99m^Tc-PYP injection. At 3 hours, neither CPV1.2 nor CPA1.2 significantly correlated with troponin T, whereas CPV1.4, CPV1.6, CPA1.4, and CPA1.6 correlated significantly and comparably with the four prognostic factors.

**Table 7:** Correlations between volumetric parameters and four prognostic factors in patients with SPECT/CT images acquired at 1 and 3 h

	LVEF (*n* = 12)		GLS (*n* = 6)		ECV (*n* = 8)		Troponin T (*n* = 9)	
	*R*^2^	*P*	*R*^2^	*P*	*R*^2^	*P*	*R*^2^	*P*
1 h								
CPV1.2	.81	< .0001*	.80	.02*	.57	.031*	.49	.036*
CPV1.4	.82	< .0001*	.81	.01*	.71	.009*	.47	.042*
CPV1.6	.75	.0003*	.85	.01*	.75	.006*	.46	.044*
CPA1.2	.83	< .0001*	.82	.01*	.63	.018*	.48	.039*
CPA1.4	.82	< .0001*	.83	.01*	.73	.007*	.46	.045*
CPA1.6	.75	.0003*	.87	.01*	.75	.006*	.45	.048*
3 h								
CPV1.2	.81	< .0001*	.71	.04*	.60	.024*	.41	.07
CPV1.4	.78	.0001*	.71	.04*	.70	.010*	.49	.03*
CPV1.6	.79	.0001*	.72	.03*	.70	.009*	.48	.04*
CPA1.2	.81	< .0001*	.70	.04*	.62	.020*	.42	.06
CPA1.4	.78	.0001*	.70	.04*	.69	.011*	.49	.04*
CPA1.6	.79	.0001*	.70	.04*	.69	.010*	.48	.04*

*CPA*, cardiac pyrophosphate activity; *CPV*, cardiac pyrophosphate volume; *ECV*, extracellular volume; *GLS*, global longitudinal strain; *LVEF*, left ventricular ejection fraction; *R*, Pearson correlation coefficient

*P < .05

## Discussion

This study has two important messages. The volumetric parameters derived from ^99m^Tc-PYP SPECT/CT, CPV and CPA quantified the ATTR-CM disease burden more accurately than SUVmax and SUVmax ratios. The performance of the volumetric parameters was equivalent between 1 and 3 hours after injecting ^99m^Tc-PYP.

Both CPV and CPA correlated more closely than the myocardial SUVmax and SUVmax ratios with all four prognostic factors (Table 3). One reason for this could be that the volumetric parameters reflect information about internal abnormal regions, whereas SUVmax and SUVmax ratios represent small areas. Furthermore, SUVmax is affected by various technical and physiological factors. Therefore, volumetric parameters could be useful imaging biomarkers for quantifying the ATTR-CM disease burden. Similarly, volumetric parameters derived from ^18^F-fluorodeoxyglucose positron emission tomography (^18^F-FDG PET) are more effective than SUVmax as prognostic predictors in cancer.^22^ Volumetric parameters were more effective than the H/CL ratio, which is valuable and widely applied, but has inherent limitations due to being based on two-dimensional images. The H/CL ratio did not correlate with LVEF, GLS, and troponin T (Table 3). Vranian et al. also showed that the H/CL ratio did not correlate with LVEF and GLS in patients with ATTR-CM.^29^ We previously showed that the H/CL ratio did not correlate with LVEF in patients with ATTR-CM.^10^

We found here that CPV and CPA significantly correlated with the four prognostic factors, LVEF, GLS, ECV, and troponin T. Therefore, CPV and CPA should play objective and important roles in prognostic evaluation. An echocardiographic LVEF reduced to < 50% predicts mortality in patients with wild type ATTR-CM (ATTRwt-CM).^25^ Furthermore, GLS is an independent predictor of all-cause mortality (hazard ratio [HR]: 1.15 per 1% decrease) and a more effective prognostic factor than all other echocardiographic parameters, including LVEF, in patients with HF and reduced LVEF.^26^ Impaired GLS is similarly a high risk factor for cardiovascular morbidity and mortality in patients with HF and preserved LVEF.^27^ Furthermore, the ECV determined by CMR has been validated for measuring the amyloid burden and it is an independent predictor of prognosis (HR: 1.155 per 3% increase).^28^ Bone scintigraphy can be an alternative modality for patients who cannot undergo contrast-enhanced CMR. High-sensitivity cardiac troponin T is used to assess prognosis in patients with ATTRwt-CM.^11,25^

Other studies similarly support the notion that ^99m^Tc-PYP scintigraphy is a useful modality for evaluating prognosis. A volumetric parameter on ^99m^Tc-PYP SPECT/CT images acquired 3 hours after injection and ECV correlated (*r*^2^ = .76, *P* = .001, *n* = 11).^7^ Even CPV and CPA derived from ^99m^Tc-PYP SPECT without CT significantly correlated with LVEF and CMR parameters, and had prognostic value and low interobserver variability.^8,9^ However, studies without CT did not correct attenuation or scatter. They acquired images only after 3 hours and applied filtered back projection. A multicenter study found that even the two-dimensional H/CL ratio of ^99m^Tc-PYP scintigraphy can deliver prognostic information.^30^

Here, we revealed for the first time that myocardial abnormal ^99m^Tc-PYP uptake quantified by SUVmax was significantly higher at 1 hour than at 3 hours (Table 4). The uptake of ^99m^Tc-DPD, which is slightly different from that of ^99m^Tc-PYP,^31^ similarly peaks at 1 hour.^20^

The present findings supported the notion that the time-efficient acquisition of ^99m^Tc-PYP SPECT images at 1 hour could accurately quantify the ATTR-CM disease burden. Correlations between prognostic factors and CPV1.4 and CPA1.4 were equivalent between 1 and 3 hours in our small cohort of patient (Table 5). Thus, larger studies are warranted. To determine whether ^99m^Tc-PYP SPECT images acquired at 1 hour are useful for prognostic evaluation is important for three reasons. Myocardial abnormal uptake peaks at 1 hour, then slowly declines. Laboratory throughput and patient satisfaction were increased by the 1-hour protocol compared with the 3-hour protocol. Some experienced centers have adopted 1-hour acquisitions, particularly in the US for ^99m^Tc-PYP imaging.^32^ A close correlation has been determined between ECV and myocardial ^99m^Tc-PYP SUVmax at 1 hour corrected for blood pool (*r*^2^ = .83, *P* = .001, *n* = 9).^12^ The diagnostic ability of ^99m^Tc-PYP three-dimensional SPECT is comparable between 1 and 3 hours.^33–35^ Delayed acquisition has been reported as a cause of false-negative diagnoses of ATTR-CM.^36^ Oncological ^18^F-FDG PET image acquisition at 1 hour is popular and delayed acquisition is optional. The expert consensus recommendation for image acquisition timing using ^99m^Tc-PYP was 1 hour in 2019.^5^ This consensus was revised to recommend image acquisition at 2 or 3 hours with 1 hour as optional. The main reason for this was to avoid false-positive diagnoses caused by excessive blood pool activity.^37^ The blood pool activity is particularly important in patients without ATTR-CM. However, the optimal method for diagnosis, including cohorts without ATTR-CM, is not necessarily the same as that for assessing disease burden,^14^ which should be assessed based on cohorts with ATTR-CM.^6,11,17–20^

We included only patients with biopsy that were positive for ATTR amyloid. This is a strength of our original study. Several studies have applied positive criteria for ATTR-CM, such as visual grading scores of 2 or 3 for myocardial uptake (≥ rib uptake). However, some patients with ATTR-CM have focal or absent ^99m^Tc-PYP uptake,^4,6,10,33^ which we identified in some of our patients with EMB-proven ATTR-CM (Figure 2B and Table 2). In addition, several causes of false-negative and false-positive results of bone scintigraphy in patients with suspected ATTR-CM have been identified.^36^

We evaluated the threshold dependence of correlations and found that the correlation coefficients of CPV1.4 and CPA1.4 were slightly closer than those of CPV1.2 and CPA1.2 for the four prognostic factors (Table 6). The optimal threshold is not necessarily identical between disease burden quantitation and diagnosis. The volumetric evaluation of bone scintigraphy could potentially be an objective marker for the diagnosis of ATTR-CM, especially in patients with focal abnormal uptake. We previously showed that CPV1.2 diagnosed ATTR-CM more effectively than CPV1.4 at 3 hours.^10^ That finding and those of images acquired at 1 and 3 hours herein (Figure 4B; Table 4) revealed that the threshold 1.4 × aortic blood pool SUVmax might result in false-negative diagnoses in patients with focal abnormal uptake that might occur during the early stage of ATTR-CM.^10^

The present study showed that the SUVmax ratios correlated more closely than myocardial SUVmax with the four prognostic factors (Table 3). This means that accurate measurements of myocardial SUVmax might not directly reflect the disease burden because myocardial SUVmax also depends on inter-organ competition for ^99m^Tc-PYP uptake. The SUV retention index, which has been mainly investigated using ^99m^Tc-DPD,^16,18,20^ has not been studied in detail using ^99m^Tc-PYP.^12^ Although the SUV retention index has been proposed to account for competition among the myocardium, bone, and soft-tissue radiotracer uptake,^16^ ^99m^Tc-DPD and ^99m^Tc-PYP uptake mechanisms slightly differ.^31^ The present study showed that the SUVmax retention index was significantly higher at 1 hour than at 3 hours. Similarly, the SUVpeak retention index using ^99m^Tc-DPD was significantly higher at 1 hour than at 3 hours.^20^

### Limitations

This retrospective analysis of a small patient cohort was conducted at a single institution. However, some of these limitations are frequent when investigating rare diseases. Furthermore, although the actual prognosis might be the ideal gold standard for prognostic studies, we did not evaluate this because of our small cohort of patients with diverse backgrounds, and the fact that some were evaluated only for short periods. Although the patients were assessed by SPECT, echocardiography, CMR, and blood tests on different days, this is unlikely to affect the conclusions because ^99m^Tc-PYP uptake does not change substantially over time.^38^ The mechanism through which bone-seeking radiotracers bind to an abnormal myocardium remains uncertain, and their relationships with the myocardial amyloid burden have not yet been histologically validated. We also need to further evaluate reproducibility and variability of volumetric parameters, which may depend on the selection of thresholds and image acquisition timing.

## Conclusions

Volumetric parameters derived from ^99m^Tc-PYP SPECT/CT, CPV and CPA quantified the ATTR-CM disease burden more accurately than the SUVmax, SUVmax ratio, and H/CL ratio. The performance was equivalent between 1 and 3 hours after injection. Larger studies are warranted to clarify an optimal nuclear imaging biomarker to manage patients with ATTR-CM.

## New Knowledge Gained

Volumetric evaluation of ^99m^Tc-PYP SPECT/CT quantified the ATTR-CM disease burden more accurately than the SUVmax and SUVmax ratios including the SUVmax retention index. Significant correlations between volumetric parameters and prognostic factors were equivalent between images acquired at 1 and 3 hours after ^99m^Tc-PYP injection, whereas the myocardial SUVmax, retention index, and visual grading score were significantly higher at 1 hour than at 3 hours.

### Supplementary Information

Below is the link to the electronic supplementary material.

Supplementary file1 (DOCX 53 kb)

Supplementary file2 (PPTX 301 kb)

## Abbreviations

ATTR-CM: Transthyretin amyloid cardiomyopathy
CPA: Cardiac pyrophosphate activity
CPV: Cardiac pyrophosphate volume
ECV: Extracellular volume
EMB: Endomyocardial biopsy
GLS: Global longitudinal strain
PYP: Pyrophosphate
SPECT: Single-photon emission computed tomography
SUV: Standardized uptake value
VOI: Volume of interest
