# Supplementary material for: Transthyretin amyloid cardiomyopathy disease burden quantified using 99mTc-pyrophosphate SPECT/CT: volumetric parameters versus SUVmax ratio at 1 and 3 hours
Source: J Nucl Cardiol. 2023 Aug 21;30(6):2721–35. doi: 10.1007/s12350-023-03353-w (PMC10682282; doi:10.1007/s12350-023-03353-w)
Supplement: Supplementary file 1 — Supplementary file1 (DOCX 53 kb) [file 12350_2023_3353_MOESM1_ESM.docx]

**Supplemental Table 1.** Correlations between uptake parameters at 1 h and four prognostic factors in patients not treated with tafamidis

|  | LVEF  (n = 18) | | GLS  (n = 13) | | ECV  (n = 11) | | Troponin T  (n = 16) | |
| --- | --- | --- | --- | --- | --- | --- | --- | --- |
|  | *R*^2^ | *P* | *R*^2^ | *P* | *R*^2^ | *P* | *R*^2^ | *P* |
| Volumetric parameters |  |  |  |  |  |  |  |  |
| CPV1.4 | 0.34 | 0.01* | 0.43 | 0.02* | 0.60 | 0.005* | 0.37 | 0.01* |
| CPA1.4 | 0.35 | 0.01* | 0.43 | 0.01* | 0.59 | 0.006* | 0.37 | 0.01* |
| SUVmax ratio |  |  |  |  |  |  |  |  |
| Myocardium/aorta | 0.08 | 0.25 | 0.17 | 0.16 | 0.52 | 0.012* | 0.22 | 0.07 |
| Myocardium/vertebra | 0.11 | 0.18 | 0.11 | 0.27 | 0.02 | 0.708 | 0.01 | 0.76 |
| Retention index^a^ | 0.26 | 0.03* | 0.25 | 0.08 | 0.01 | 0.748 | 0.02 | 0.64 |
| Myocardial SUVmax | 0.00 | 0.94 | 0.01 | 0.73 | 0.20 | 0.168 | 0.00 | 0.90 |
| H/CL ratio | 0.14 | 0.13 | 0.07 | 0.38 | 0.61 | 0.005* | 0.20 | 0.08 |
| Visual grading score | 0.04 | 0.40 | 0.10 | 0.30 | -^b^ | -^b^ | 0.04 | 0.47 |

*CPA*, cardiac pyrophosphate activity; *CPV*, cardiac pyrophosphate volume; *ECV*, extracellular volume fraction; *GLS*, global longitudinal strain; *H/CL*, heart to contralateral lung; *LVEF*, left ventricular ejection fraction; *R*, correlation coefficient; *SUV*, standardized uptake value

**P* < 0.05.

^a^ Retention index = (myocardial SUV/vertebral SUV) × paraspinal-muscle SUV.

^b^ Visual grading score of all 11 patients were 3.

**Supplemental Table 2.** Correlations between uptake parameters at 1 h and four prognostic factors

|  | LVEF  (n = 22) | | GLS  (n = 13) | | ECV  (n = 12) | | Troponin T  (n = 18) | |
| --- | --- | --- | --- | --- | --- | --- | --- | --- |
|  | *R*^2^ | *P* | *R*^2^ | *P* | *R*^2^ | *P* | *R*^2^ | *P* |
| CPV1.4 | 0.45 | 0.0006* | 0.43 | 0.02* | 0.63 | 0.002* | 0.42 | 0.004* |
| CPV1.4_LV_^a^ | 0.46 | 0.0006* | 0.45 | 0.01* | 0.57 | 0.005* | 0.38 | 0.006* |

*CPV*, cardiac pyrophosphate volume; *ECV*, extracellular volume fraction; *GLS*, global longitudinal strain; *LV*, left ventricle; *LVEF*, left ventricular ejection fraction; *R*, Pearson correlation coefficient

**P* < 0.05.

^a^ CPV1.4_LV_ is a CPV1.4 evaluated using only the left ventricular region and excluding right ventricular activity.

**Supplemental Table 3.** Correlations between uptake parameters at 1 h and eGFR

|  | eGFR  (n = 23) | |
| --- | --- | --- |
|  | *R*^2^ | *P* |
| Volumetric parameters |  |  |
| CPV1.4 | 0.01 | 0.74 |
| CPA1.4 | 0.00 | 0.75 |
| SUVmax ratio |  |  |
| Myocardium/aorta | 0.02 | 0.51 |
| Myocardium/vertebra | 0.06 | 0.28 |
| Retention index^a^ | 0.06 | 0.27 |
| Myocardial SUVmax | 0.00 | 0.89 |
| H/CL ratio | 0.13 | 0.09 |
| Visual grading score | 0.13 | 0.09 |

*CPA*, cardiac pyrophosphate activity; *CPV*, cardiac pyrophosphate volume; *eGFR*, estimated glomerular filtration rates; *R*, correlation coefficient; *SUV*, standardized uptake value

^a^ Retention index = (myocardial SUV/vertebral SUV) × paraspinal-muscle SUV.


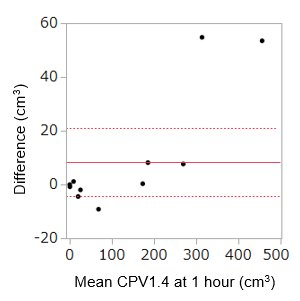


**Supplemental Figure 1.** Bland-Altman plot showing the interobserver variability in cardiac pyrophosphate volume (CPV) 1.4 measurement at 1 hour (n = 13).
