# Supplementary material for: Transthyretin amyloid cardiomyopathy disease burden quantified using 99mTc-pyrophosphate SPECT/CT: volumetric parameters versus SUVmax ratio at 1 and 3 hours
Source: J Nucl Cardiol. 2023 Aug 21;30(6):2721–35. doi: 10.1007/s12350-023-03353-w (PMC10682282; doi:10.1007/s12350-023-03353-w)
Supplement: Supplementary file 2 — Supplementary file2 (PPTX 301 kb) [file 12350_2023_3353_MOESM2_ESM.pptx]

## Slide 1
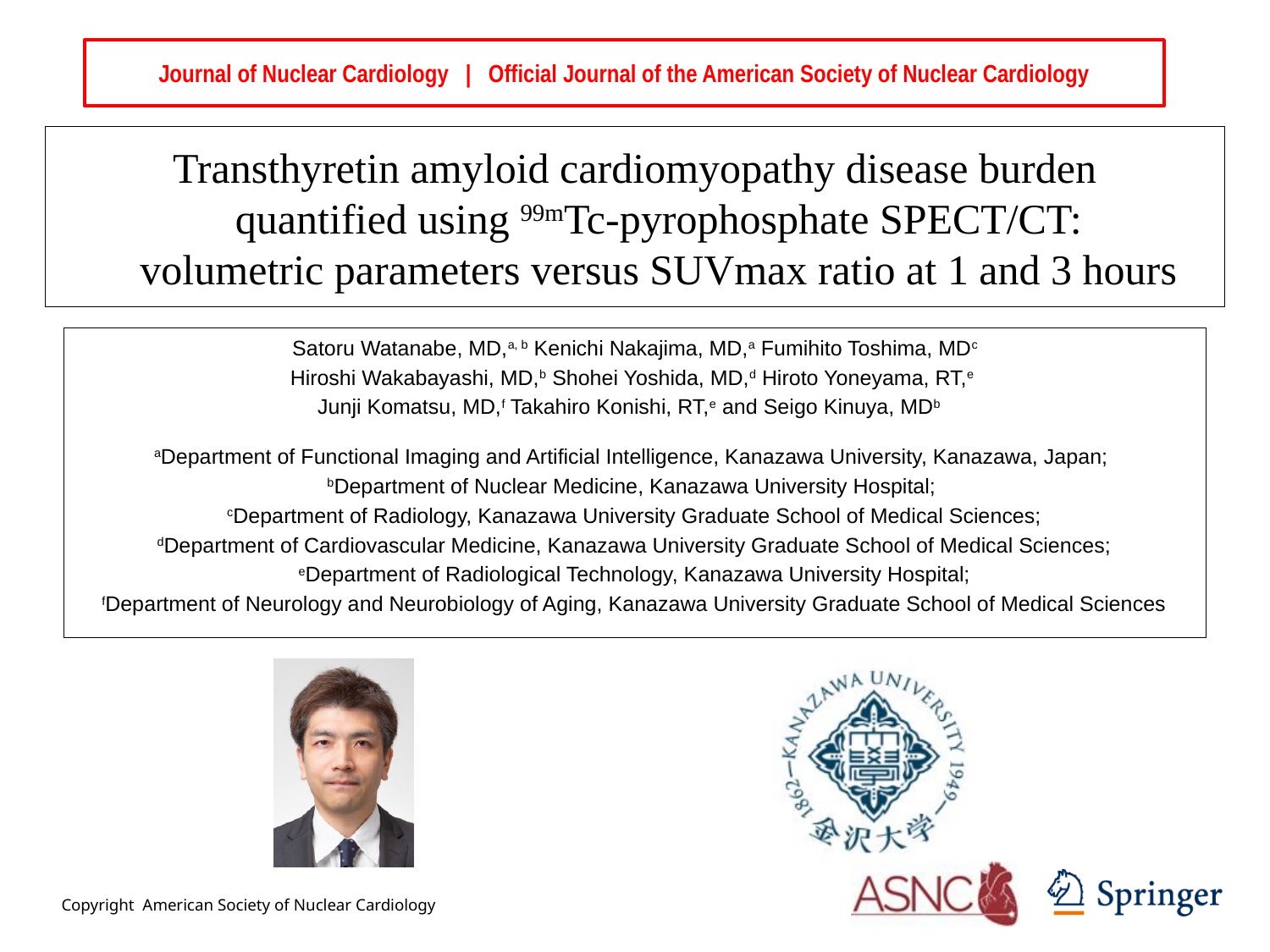

Journal of Nuclear Cardiology | Official Journal of the American Society of Nuclear Cardiology
# Transthyretin amyloid cardiomyopathy disease burden quantified using 99mTc-pyrophosphate SPECT/CT: volumetric parameters versus SUVmax ratio at 1 and 3 hours
Satoru Watanabe, MD,a, b Kenichi Nakajima, MD,a Fumihito Toshima, MDc
Hiroshi Wakabayashi, MD,b Shohei Yoshida, MD,d Hiroto Yoneyama, RT,e
Junji Komatsu, MD,f Takahiro Konishi, RT,e and Seigo Kinuya, MDb
aDepartment of Functional Imaging and Artificial Intelligence, Kanazawa University, Kanazawa, Japan;
bDepartment of Nuclear Medicine, Kanazawa University Hospital;
cDepartment of Radiology, Kanazawa University Graduate School of Medical Sciences;
 dDepartment of Cardiovascular Medicine, Kanazawa University Graduate School of Medical Sciences;
eDepartment of Radiological Technology, Kanazawa University Hospital;
fDepartment of Neurology and Neurobiology of Aging, Kanazawa University Graduate School of Medical Sciences
Copyright American Society of Nuclear Cardiology

## Slide 2
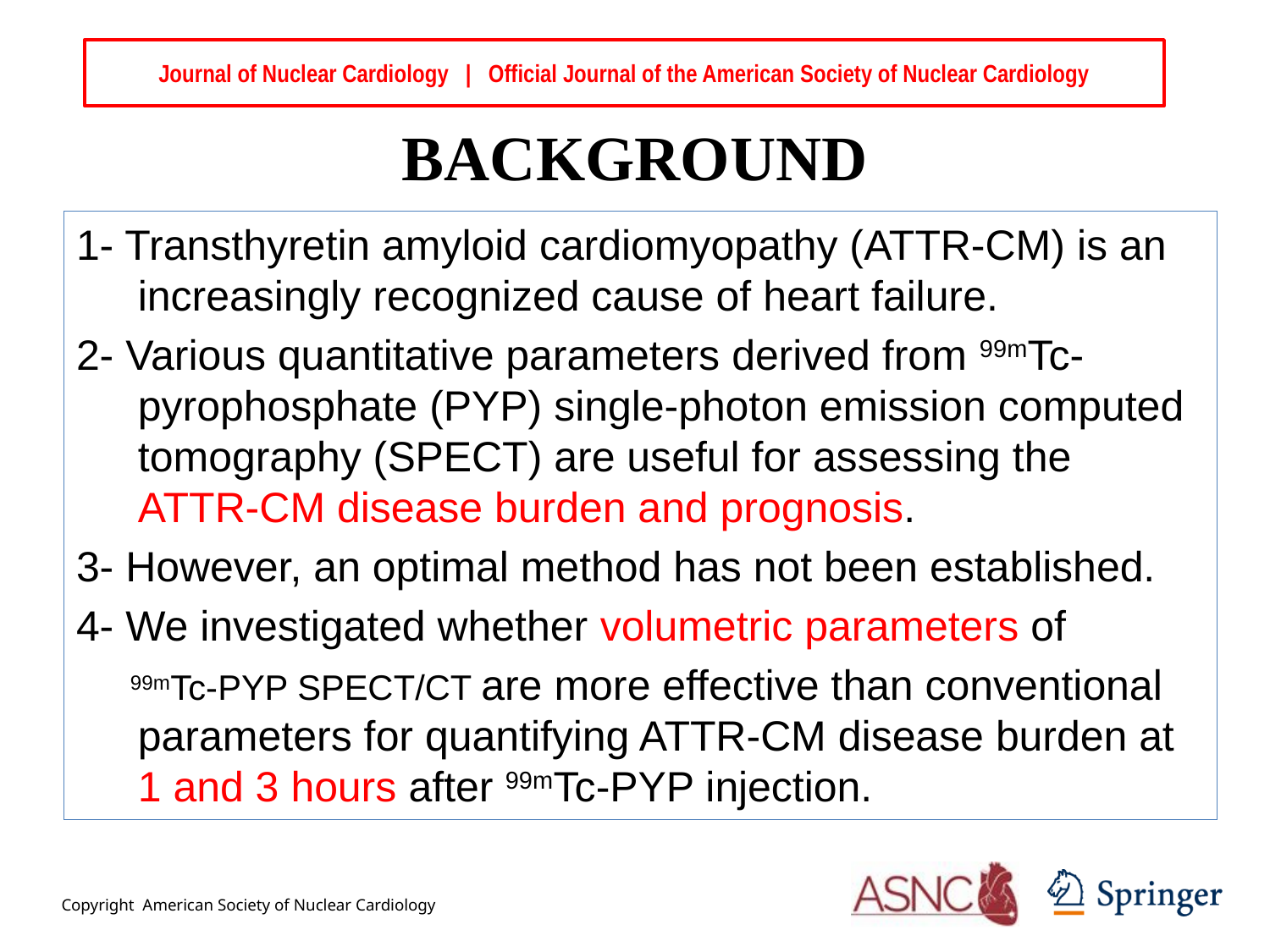

Journal of Nuclear Cardiology | Official Journal of the American Society of Nuclear Cardiology
# BACKGROUND
1- Transthyretin amyloid cardiomyopathy (ATTR-CM) is an increasingly recognized cause of heart failure.
2- Various quantitative parameters derived from 99mTc-pyrophosphate (PYP) single-photon emission computed tomography (SPECT) are useful for assessing the ATTR-CM disease burden and prognosis.
3- However, an optimal method has not been established.
4- We investigated whether volumetric parameters of
 99mTc-PYP SPECT/CT are more effective than conventional parameters for quantifying ATTR-CM disease burden at 1 and 3 hours after 99mTc-PYP injection.
Copyright American Society of Nuclear Cardiology

## Slide 3
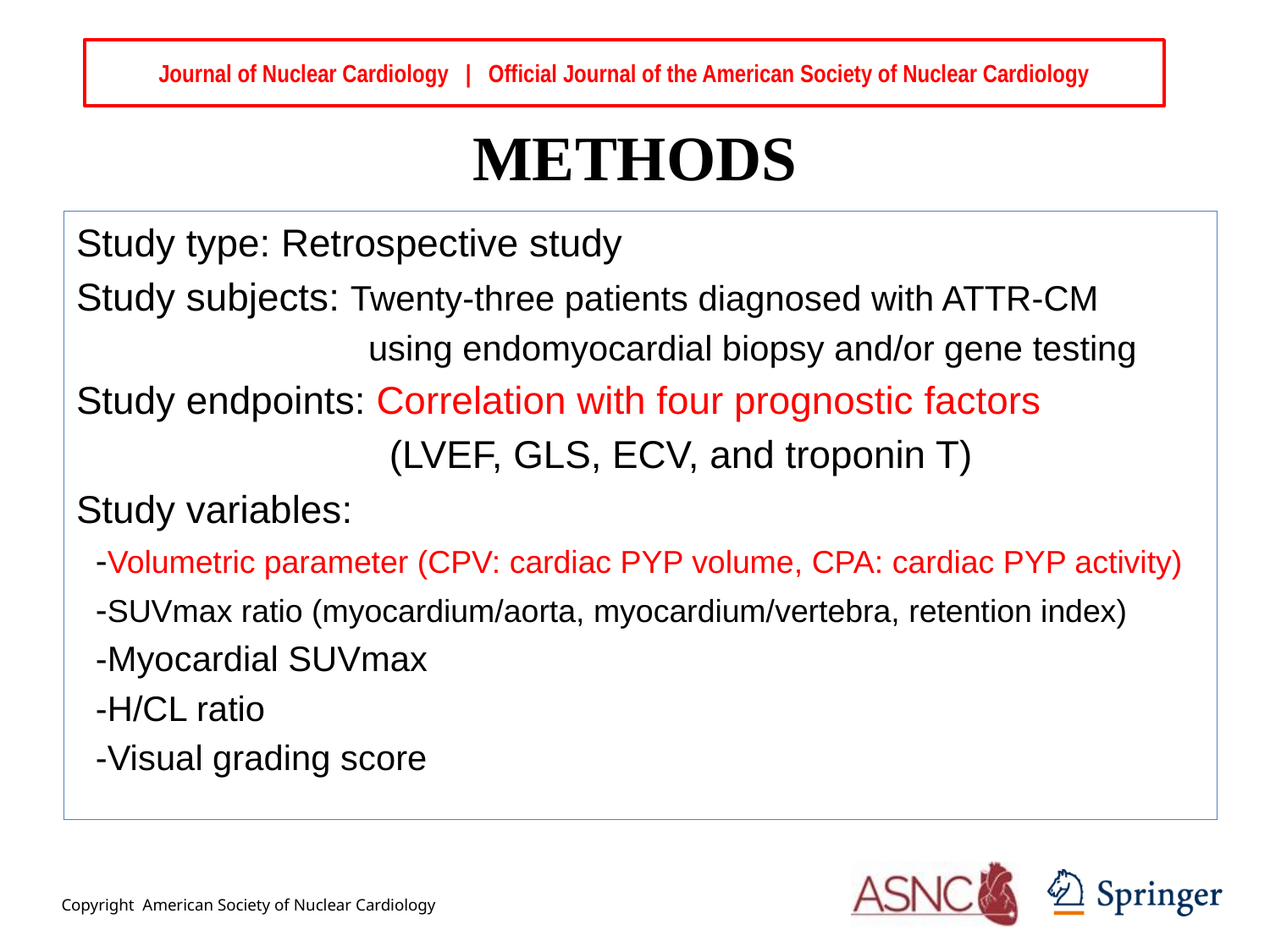

Journal of Nuclear Cardiology | Official Journal of the American Society of Nuclear Cardiology
# METHODS
Study type: Retrospective study
Study subjects: Twenty-three patients diagnosed with ATTR-CM
 using endomyocardial biopsy and/or gene testing
Study endpoints: Correlation with four prognostic factors
 (LVEF, GLS, ECV, and troponin T)
Study variables:
 -Volumetric parameter (CPV: cardiac PYP volume, CPA: cardiac PYP activity)
 -SUVmax ratio (myocardium/aorta, myocardium/vertebra, retention index)
 -Myocardial SUVmax
 -H/CL ratio
 -Visual grading score
Copyright American Society of Nuclear Cardiology

## Slide 4
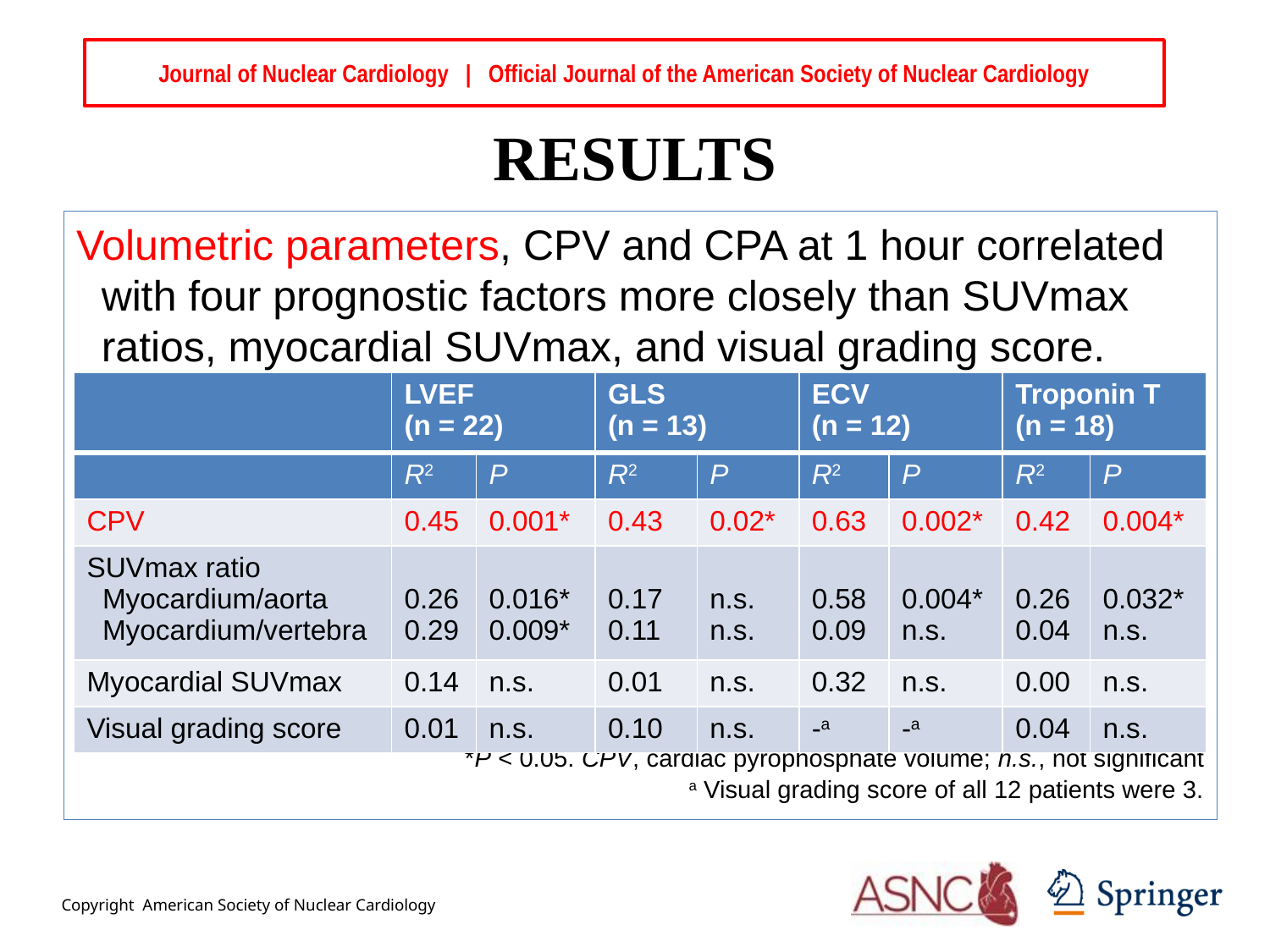

Journal of Nuclear Cardiology | Official Journal of the American Society of Nuclear Cardiology
# RESULTS
Volumetric parameters, CPV and CPA at 1 hour correlated with four prognostic factors more closely than SUVmax ratios, myocardial SUVmax, and visual grading score.
*P < 0.05. CPV, cardiac pyrophosphate volume; n.s., not significant
a Visual grading score of all 12 patients were 3.
| | LVEF (n = 22) | | GLS (n = 13) | | ECV (n = 12) | | Troponin T (n = 18) | |
| --- | --- | --- | --- | --- | --- | --- | --- | --- |
| | R2 | P | R2 | P | R2 | P | R2 | P |
| CPV | 0.45 | 0.001\* | 0.43 | 0.02\* | 0.63 | 0.002\* | 0.42 | 0.004\* |
| SUVmax ratio Myocardium/aorta Myocardium/vertebra | 0.26 0.29 | 0.016\* 0.009\* | 0.17 0.11 | n.s. n.s. | 0.58 0.09 | 0.004\* n.s. | 0.26 0.04 | 0.032\* n.s. |
| Myocardial SUVmax | 0.14 | n.s. | 0.01 | n.s. | 0.32 | n.s. | 0.00 | n.s. |
| Visual grading score | 0.01 | n.s. | 0.10 | n.s. | -a | -a | 0.04 | n.s. |
Copyright American Society of Nuclear Cardiology

## Slide 5
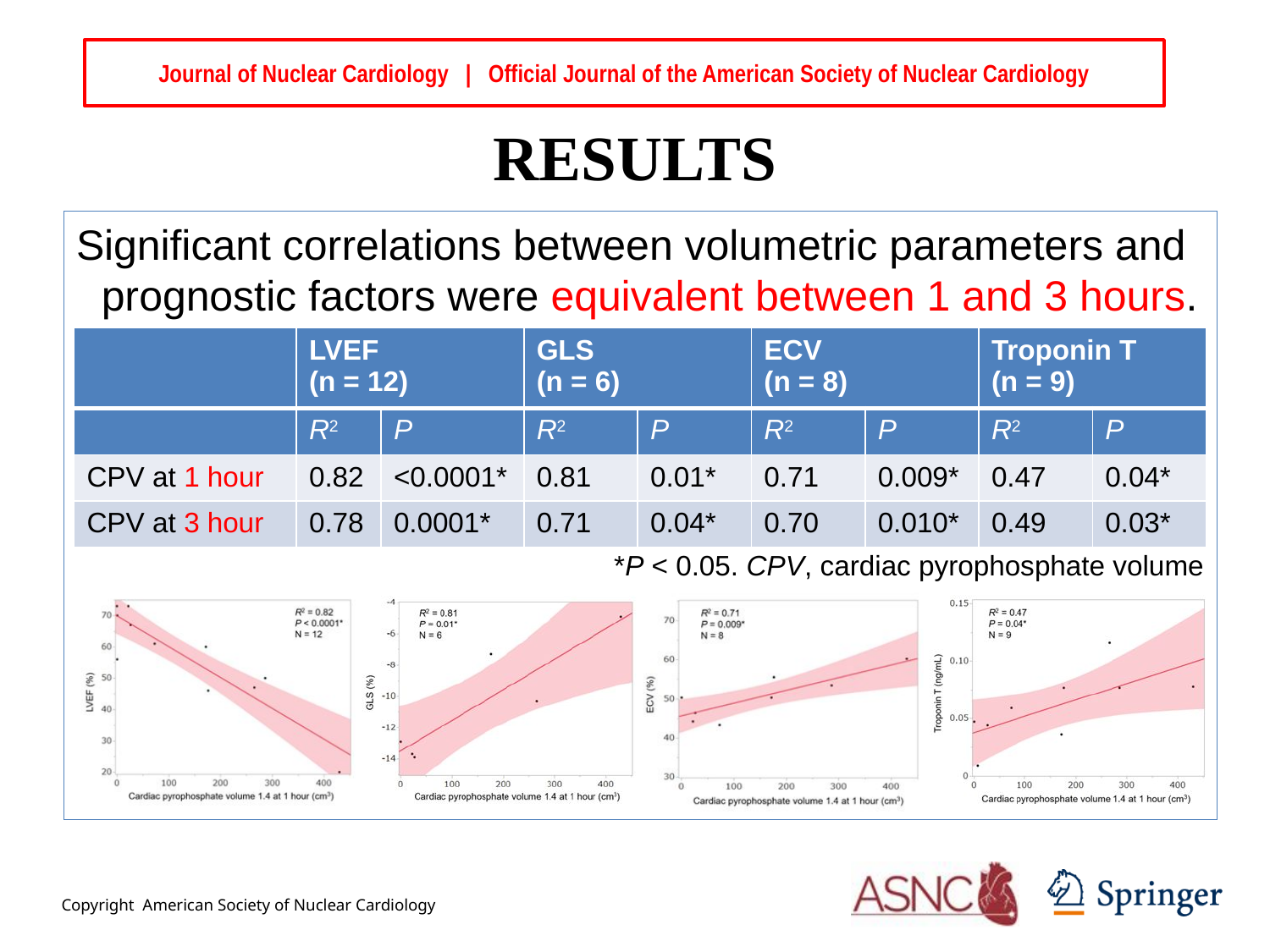

Journal of Nuclear Cardiology | Official Journal of the American Society of Nuclear Cardiology
# RESULTS
Significant correlations between volumetric parameters and prognostic factors were equivalent between 1 and 3 hours.
 *P < 0.05. CPV, cardiac pyrophosphate volume
| | LVEF (n = 12) | | GLS (n = 6) | | ECV (n = 8) | | Troponin T (n = 9) | |
| --- | --- | --- | --- | --- | --- | --- | --- | --- |
| | R2 | P | R2 | P | R2 | P | R2 | P |
| CPV at 1 hour | 0.82 | <0.0001\* | 0.81 | 0.01\* | 0.71 | 0.009\* | 0.47 | 0.04\* |
| CPV at 3 hour | 0.78 | 0.0001\* | 0.71 | 0.04\* | 0.70 | 0.010\* | 0.49 | 0.03\* |
Copyright American Society of Nuclear Cardiology

## Slide 6
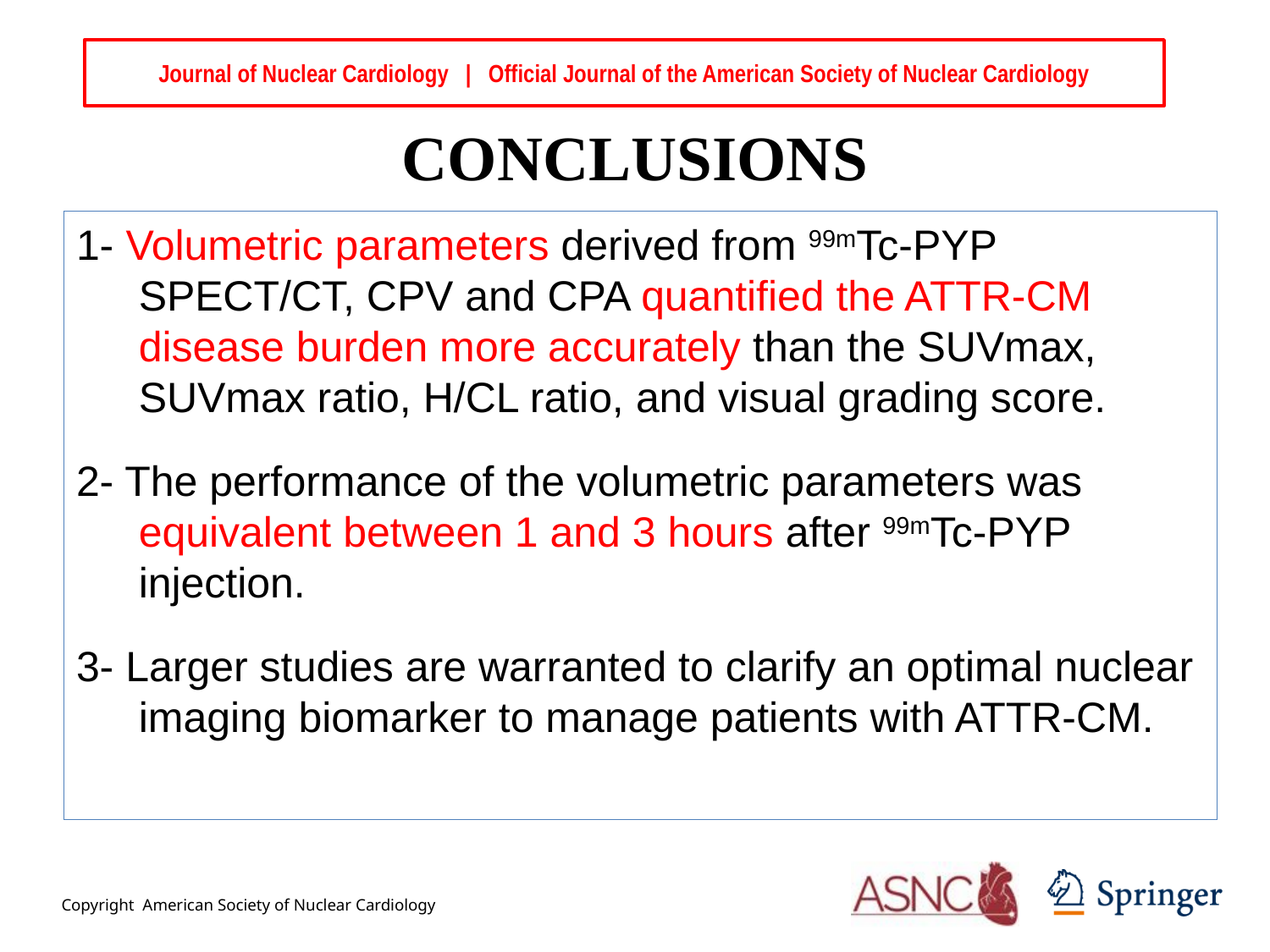

Journal of Nuclear Cardiology | Official Journal of the American Society of Nuclear Cardiology
# CONCLUSIONS
1- Volumetric parameters derived from 99mTc-PYP SPECT/CT, CPV and CPA quantified the ATTR-CM disease burden more accurately than the SUVmax, SUVmax ratio, H/CL ratio, and visual grading score.
2- The performance of the volumetric parameters was equivalent between 1 and 3 hours after 99mTc-PYP injection.
3- Larger studies are warranted to clarify an optimal nuclear imaging biomarker to manage patients with ATTR-CM.
Copyright American Society of Nuclear Cardiology
